# Clinical Applicability of a Terminology Subset for Monitoring Child Development from Zero Months to Age Three: An Observational Study

**DOI:** 10.3390/ijerph23081048

**Published:** 2026-08-12

**Authors:** Mariane Mota Dhein, Soraia Matilde Marques Buchhorn, Maria de La Ó Ramallo Veríssimo, Francine Dutra Mattei, Sabrina do Rocio Kopietz Cremonez, Marcia Regina Cubas

**Affiliations:** 1 Programa de Pós-Graduação em Tecnologia em Saúde, Pontifícia Universidade Católica do Paraná, Curitiba 80215-901, PR, Brazil; m.cubas@pucpr.br; 2 Departamento de Enfermagem em Saúde Coletiva, Universidade Federal de São Paulo, São Paulo 01246-900, SP, Brazil; soraia.buchhorn@unifesp.br; 3 Departamento de Enfermagem Materno-Infantil e Psiquiátrica, Universidade de São Paulo, São Paulo 05403-000, SP, Brazil; mdlorver@usp.br; 4 Curso de Enfermagem, Pontifícia Universidade Católica do Paraná, Curitiba 80215-901, PR, Brazil; francine.mattei@pucpr.br; 5 Hospital e Maternidade Nossa Senhora de Fátima, Unimed Curitiba, Curitiba 80010-100, PR, Brazil; sa_kopietz@hotmail.com

**Keywords:** nursing diagnosis, nursing outcomes, nursing interventions, community health nursing

## Abstract

**Highlights:**

**Public health relevance—How does this work relate to a public health issue?**

- The relevance of this study to public health is linked to the fact that studying child development in the context of nursing consultations is essential to achieving high-quality monitoring of growth and development, as well as measuring the positive impact achieved.

**Public health significance—Why is this work of significance to public health?**

- The results demonstrate that the bond between the nurse and the child’s caregiver is crucial for ensuring consistent attendance at consultations.
- The quality of care, as well as the documentation of care, is related to the nurse’s concern with the longitudinality of the care provided to the developing child.

**Public health implications—What are the key implications or messages for practitioners, policy makers and/or researchers in public health?**

- Evaluating the clinical applicability of the subset in a real-world care practice context provides data on the representativeness of nursing diagnoses, outcomes, and interventions in primary health care (PHC) child care. Given that monitoring child development is a priority in Primary Health Care, having a subset capable of standardizing what the nurse diagnoses, performs, and achieves in terms of outcomes can foster more accurate care with data that enables measuring the impact of the interventions carried out by the nurse.

**Abstract:**

Child development monitoring by nurses enables the identification of alterations and early interventions. This study analyzed the clinical applicability of a terminology subset for monitoring the development of children aged zero to three years during nursing consultations. This is a cross-sectional, observational study that analyzed the applicability of a terminology subset. Three stages of the clinical applicability analysis method for terminology subsets were followed: development of the data collection instrument; systematic observation of 46 consultations in two Brazilian municipalities; and data organization and analysis. Nursing diagnoses, outcomes, and interventions observed at least once during a consultation were considered applicable. Of the analyzed subset, 64.7% of the diagnoses/outcomes and 21.3% of the interventions were observed during consultations and are applicable to clinical practice. Notably, the diagnosis “Child development, effective” was identified in 93% of the consultations. The data suggest the applicability of the subset in specific Brazilian primary healthcare contexts, predominantly with healthy children.

## 1. Introduction

Brazilian public policies aimed at child healthcare define child development as a complex, continuous, dynamic, and progressive transformation that, in addition to growth, encompasses maturation, learning, and the child’s psychological and social aspects [1]. It is argued that the child development process is interconnected with physical, environmental, social, educational, and health aspects [1].

Child development surveillance, follow-up, and monitoring actions are essential to promoting a child’s quality of life. They enable early identification of developmental changes and delays, allowing for interventions that minimize future harm to this population [2,3]. These actions are strategically carried out in Primary Health Care (PHC) through well-child visits [3]. As members of the healthcare team, nurses act in child health care and conduct nursing consultations aligned with international approaches to promote early childhood development.

In Brazil, the nursing consultation is regulated and organized by the steps of the Nursing Process, which comprise: nursing assessment, nursing diagnosis, nursing planning, nursing implementation, and nursing evaluation [4].

For the documentation of nursing consultations, the use of standardized terminology is recommended. One such terminology is the International Classification for Nursing Practice (ICNP^®^), developed since 1989 by the International Council of Nurses (ICN) and represented, since 2021, by Systematized Nomenclature of Medicine—Clinical Terms (SNOMED CT). SNOMED CT is a clinically validated, multilingual vocabulary that enables the standardization of documentation and global information sharing among healthcare professionals by integrating different classification systems [5,6,7,8]. The inclusion of nursing terminologies in SNOMED CT will allow the retrieval of information related to nursing actions, enabling the analysis of their impact on population health outcomes [9]. Therefore, research focusing on standardized terminologies is relevant to support the representation of nursing practices in interoperable systems.

ICNP^®^ nursing diagnoses, outcomes, and interventions can be organized into terminology subsets aimed at a specific specialty, clientele, or nursing phenomenon. Subsets serve as structuring tools for professional practice, as they enhance the clinical decision-making process, support systemized care documentation, and favor the delivery of individualized, organized care centered on the needs of the individual, family, or community [10,11,12].

In the current context, where the integration of ICNP^®^ into SNOMED CT expands the possibilities for interoperability of nursing records [9], analyzing the clinical applicability of subsets as a technology for documenting practices and supporting professional decision-making becomes highly relevant.

The terminology subset “Assessment/monitoring of child development in children aged zero to three years” was published as an integral part of a doctoral dissertation in 2014 [13]. The subset was grounded in frameworks of comprehensive assessment and human development, and incorporated developmental milestones used in the “Child Health Booklet” (Caderneta de Saúde da Criança), an instrument adopted in Brazilian PHC [9].

The theoretical frameworks used were Bronfenbrenner’s bioecological model [14] and Brazelton and Greenspan’s essential needs of children [15]. The former asserts that human development results from the interaction of four elements: (a) person; (b) time; (c) context: factors that directly or indirectly interfere with development, such as home, school, and community; and (d) process: reciprocal interactions between the human being and other people, such as interactions with parents, caregivers, and other children [13]. The latter posits that children have needs for: (a) ongoing nurturing relationships—human interaction with affection; (b) physical protection, safety, and regulation—physical and physiological integrity; (c) experiences tailored to individual differences—temperament, skills, and competencies; (d) developmentally appropriate experiences—according to each age group; (e) limited setting, structure, and expectations—discipline as an affectionate teaching–learning process that encourages children to pursue their goals; and (f) stable, supportive communities and cultural continuity—a support network within the community and society [14].

The aforementioned terminology subset underwent a content validation stage, which refers to expert opinion [13].

To enhance consistency in the use of the subset, it is important to analyze whether it is applicable in clinical nursing practice. Studies on the applicability analysis of subsets are scarce, and an unsystematic literature search in databases yielded no applicability studies in PHC. Two studies were identified that analyzed the clinical applicability of terminology subsets in hospital settings: one validated nursing diagnoses, outcomes, and interventions for palliative care patients in a hospital and hospice [16], and the other validated nursing interventions for postpartum women [17].

This study fills the gap regarding the clinical applicability of subsets within the context of PHC and contributes to the refinement of the applicability analysis method proposed by Cubas and collaborators [18].

Given this context, the objective of this study was to analyze the clinical applicability of the terminology subset for monitoring the development of children aged zero to three years.

## 2. Materials and Methods

This is an observational, cross-sectional study with a quantitative approach that employed the stages of the clinical applicability analysis method for terminology subsets in Primary Health Care (PHC) [18]. This method comprises three prerequisites and four stages. The prerequisites were: selection of research sites, selection of participants, and the nurses’ knowledge regarding the subset. The stages consisted of: development of the data collection instrument, observation of the nursing consultation, and data organization and analysis. The stage involving the observation of a follow-up consultation, which is originally foreseen in the method, was not conducted due to scheduling constraints.

The empirical base used was the “ICNP^®^ Subset for monitoring child development in children aged zero to three years,” consisting of 17 nursing diagnoses/outcomes and 164 nursing interventions [13].

### 2.1. Research Site

The study was conducted in three Basic Health Units (BHUs) in Brazil: two BHUs located in the state of Paraná (Southern region of the country) and one in the state of São Paulo (Southeastern region). The Paraná BHUs are located in a municipality within the metropolitan area of the state capital. The first unit covers an area of approximately 560 m^2^, serves a population of about 37,000 users, and has a team of 50 professionals, including physicians, nurses, dentists, psychologists, nutritionists, physical therapists, pharmacists, and nursing technicians, as well as community health workers. The second unit measures approximately 332 m^2^, serves a population of about 19,000 users, and its team consists of 32 professionals, including physicians, nurses, nursing technicians, dentists, oral health technicians, psychologists, nutritionists, and physical therapists. The São Paulo BHU is located in the state capital, serves a population of approximately 29,000 users, and has a team of 120 professionals, including physicians, nurses, nursing technicians, dentists, psychologists, nutritionists, speech–language pathologists, social workers, physical therapists, physical educators, and occupational therapists. All units are supported by administrative and cleaning teams.

### 2.2. Participants

The applicability analysis method recommends observing at least 20 consultations [15], without sample size calculation. In the state of Paraná, the researcher observed consultations until reaching the minimum recommended number, attending the BHU weekly over a six-month period. In the state of São Paulo, it was possible to observe six consultations beyond the recommended minimum, as these consultations occurred on the same day the minimum threshold was reached. At this site, the researcher attended the BHU weekly over an eight-month period. Thus, a total of 46 consultations were observed.

Participants were divided into two groups: (G1) 46 children aged zero to three years and their primary caregivers, with 20 recruited in Paraná and 26 in São Paulo, and (G2) eight nurses, three from Paraná and five from São Paulo. The inclusion criteria for G1 were children of both sexes, aged zero to three years, undergoing well-child care follow-up at the BHU, and accompanied by a legal guardian or caregiver aged 18 years or older at the time of the nursing consultation. Children whose caregivers presented any temporary or chronic communication difficulty were excluded. The inclusion criteria for G2 were a minimum of two years of clinical experience and the routine performance of well-child consultations in their healthcare practice. Nurses who had not conducted well-child consultations as part of their routine in the past six months were excluded. Participant recruitment was conducted through direct invitation by the researchers in the waiting room of the health unit, via access to routine child health visits. There were no refusals to participate in the study or losses of nurse participants.

### 2.3. Data Collection, Organization and Analysis Procedures

Prior to the start of data collection, the following procedures were conducted: (a) clarification of doubts and calibration of the two nurse observers regarding the data collection procedure and instrument use, conducted virtually; (b) clarification of any questions from the nurses and request for authorization to observe consultations upon signing the Informed Consent Form; (c) provision of a printed version of the terminology subset to the nurses, followed by a discussion on its content; (d) selection of children according to the BHU schedule, jointly with the nurse responsible for the consultation; (e) verification of the inclusion and exclusion criteria for both the child and the caregiver; and (f) explanation of the study to the caregiver and request for authorization for data collection upon signing the Informed Consent Form.

The two data collection instruments were developed exclusively for this study and refined through a pilot test. The first instrument contained data for characterizing the nurse who conducted the consultation and initial assessment data of the child. The second instrument was designed according to the steps of the Nursing Process, based on the terminology subset for monitoring child development in children aged zero to three years [13] and on the Child Health Booklet (Caderneta da Criança) [19]. This instrument included data regarding: age in months (zero to 36 months); developmental milestones; general nursing diagnoses/outcomes; 17 nursing diagnoses/outcomes categorized by domains (motor, language, adaptive, and personal–social); general interventions; and interventions categorized by domains (motor, language, adaptive, and personal–social).

The systematic observation of the nursing consultation was conducted without any interference from the researcher-observer. Each observation lasted for the duration of the consultation performed by the professional, averaging 30 min. No observed consultation was excluded, as all of them included the assessment of child development.

“Identified nursing diagnosis/outcome” was defined as the diagnosis or outcome identified by the nurse during the consultation, regardless of whether it was recorded in the Electronic Health Record. “Observed nursing diagnosis/outcome” referred to the diagnoses or outcomes identified by the researcher during the consultation. “Prescribed interventions” were those recorded by the nurse in the Electronic Health Record, whereas “observed interventions” were those identified by the researcher. After the end of each consultation, the nurse was asked to check the collected data in order to prevent potential observer misinterpretations.

Medical record data were transcribed into the data collection instrument by the primary researcher and verified by the nurse responsible for the consultation. The observed and verified data were transferred to a Microsoft Office Excel^®^ spreadsheet by the primary researcher with the assistance of an undergraduate research scholar. Data were analyzed individually per consultation.

Data analysis was performed using simple descriptive statistics, and the findings were discussed according to the theoretical framework. Nursing diagnoses, outcomes, and interventions were considered applicable in clinical nursing practice if they had been identified by the nurses or observed by the observer at least once, at any time during the observed consultations, in accordance with the concept of applicability specified by the method used [18]. That is, nursing diagnoses, outcomes, and interventions were judged by the researchers to be applicable if they had been identified or observed at least once at any point in any consultation, in line with the reference method used.

### 2.4. Ethical Considerations

The study was approved by the Research Ethics Committees of the Pontifícia Universidade Católica do Paraná (PUCPR) under opinion number CAAE 56765322.1.1001.0020; of the Municipal Health Secretariat of São José dos Pinhais under opinion number CAAE 5675322.1.3001.0086; and of the Municipal Health Secretariat of São Paulo under opinion number CAAE 5675322.1.2001.5505. The identities of all participants were omitted, thereby ensuring anonymity in both groups. The data for this study are accessible via the following link: https://doi.org/10.5281/zenodo.20797901.

## 3. Results

A total of 46 nursing consultations performed with children were observed; of these, 26 children were female and 20 were male. Regarding the distribution by age group in months, the categories were: up to one month (3 children), two to four months (18 children), four to six months (11 children), nine to 12 months (7 children), 12 to 15 months (2 children), 18 to 24 months (2 children), and 24 to 30 months (1 child). During the data collection period, no consultations were conducted for children aged 6 to 9 months or 15 to 18 months in either study site. These children are socioeconomically vulnerable and predominantly rely on the public healthcare system (a free service in the country).

Regarding the eight participating nurses, seven were female and one was male, with clinical practice experience ranging from eight to eighteen years. In their routine practice, they had been conducting childcare consultations for a minimum of two and a maximum of nine years, and had been working in PHC between one and ten years.

Of the 17 nursing diagnoses/outcomes included in the subset, 11 were identified or observed during data collection, consisting of six positive diagnoses/outcomes and five risk-related ones (Table 1).

The following nursing diagnoses/outcomes were not identified: impaired child development; impaired psychomotor activity; Impaired communication; risk for impaired cognitive learning; impaired cognitive learning; and impaired personal–social ability.

The diagnoses, categorized by child development domain, are presented in Table 2.

In the 46 observed nursing consultations, a total of 673 general nursing interventions were performed (unique or repeated), with an average of 14.63 nursing interventions per consultation. Of the 164 nursing interventions included in the subset, only 13 nursing interventions were identified and observed, which recurred across multiple consultations. The nursing interventions identified or observed in more than 20 consultations are described in Table 3.

The nursing interventions identified or observed in fewer than 20 consultations were: providing anticipatory guidance on child development characteristics; motivating the mother to stimulate child development; promoting a safe and stimulating environment for the child; Reviewing health history; and ensuring continuity of care.

## 4. Discussion

This study evaluated the clinical applicability of a terminological subset for monitoring the child development of children aged zero to three years during childcare consultations in PHC. The observed coverage for nursing diagnoses/outcomes (64.7%) suggests that a substantial portion of these subset elements is activated in real-world consultations, especially the nursing diagnoses/outcomes focused on child development promotion and overall assessment. The high frequency of the nursing diagnosis “Child development, effective” (93.5%) indicates a predominance of assessments classified as appropriate within the observed scenarios. This is compatible with routine childcare consultations, which focus on disease prevention and health promotion actions.

This finding aligns with results reported by Costa et al. (2018) [20] in São Paulo, Brazil, which analyzed the nursing diagnoses listed in newborn consultations. The nursing diagnosis “Newborn development, effective” was identified in 100% of the medical records across the 37 newborns monitored. It is plausible to consider that the nursing diagnosis “Child development, effective” is not only applicable to the clinical practice of nurses conducting childcare consultations but is also a consolidated nursing diagnosis. Within the ICNP^®^, it has been included as a diagnostic concept since the 2011 version. This finding confirms that nurses utilize and document the nursing diagnosis “Child development, effective,” thereby enabling the analysis of the impact of child development-promoting actions and outcomes sensitive to nursing interventions.

Despite the advances in public policies focused on child health and development, regional inequalities persist that limit their impact. Between 2010 and 2022, infant mortality in Brazil showed a slight reduction, from 13.0 to 12.7 deaths per 1000 live births, with lower rates concentrated in the Southern and Southeastern regions. Conversely, the Northern and Northeastern regions accounted for 42.2% and 28.2% of deaths, respectively [21]. In this sense, overcoming strategies are necessary, particularly those aimed at expanding access to consultations that identify risk situations and propose interventions with the potential to transform reality. The analyzed subset can be considered an instrument with such potential.

Another result to be discussed relates to the nursing diagnosis “Caregiver attention,” associated with the nursing interventions “Inform mother/caregiver about evaluation result” and “Praise mother/caregiver.” The identification of these elements demonstrates the importance of parental or guardian participation in child care. Authors who analyzed the quality of child PHC in northeastern Brazil argue that family involvement during well-child consultations is essential for monitoring child development. They emphasize that the family must be empowered during the consultation, given that this central unit is capable of making health-promoting choices and recognizing warning signs related to child development [22].

It is worth noting that the frequency of positive statements—both general and by specific domain—demonstrates that nurses identify child health promotion situations in their care routine; that is, their clientele is eminently healthy. A study analyzing factors that influence nursing practice in well-child care discussed that the nurse’s perspective on child health promotion provides comprehensive care to the child [23]. Conversely, the results of a systematic literature review analyzing evidence on the clinical competencies of pediatric nurses concluded that, although competencies for health education, counseling, child health and development assessment, immunizations, and child health screenings were identified, no evidence of autonomously performed nursing practices was found [24]. In other words, although the nurse’s promotional vision is identified as relevant, there is a need to investigate more deeply the impact of autonomous nursing care on child health promotion. Thus, retrieving information from nursing records using standardized terminologies can assist in analyzing outcomes sensitive to nursing actions in the context of child development.

Another notable result involves the participating nurses’ concern with continuity of care, which was identified through the prescription and documentation of the nursing interventions “Plan follow-up consultation for child development evaluation,” “Document child information,” and “Apply child development evaluation instrument.” It is important to emphasize that quality of care is inherently related to continuity of care and the professional–patient bond. This aspect was analyzed by a study conducted in northeastern Brazil, which identified that 80% of family health teams maintained a bond with the child and their family; that is, the same professional followed the child over time [22]. Therefore, continuity of care fosters mutual trust and offers opportunities for health promotion and risk prevention related to child development.

This bond is related to the dialogue between the nurse and the child’s caregiver. The importance of dialogue with parents or caregivers during child development evaluation was demonstrated by the presence of the nursing interventions “Reinforce with mother/caregiver how to stimulate the child” and “Inform mother/caregiver about the importance of play for the child.” Studies involving children of other age groups or health conditions highlight the relevance of dialogue and corroborate the findings presented in this study. Results of a review identifying strategies used by nurses in caring for children and adolescents with autism indicate that active parental involvement is the second most mentioned strategy by nurses. These professionals emphasize that the action plan must be evaluated and shared with parents to achieve the best outcome for the child [25].

It is well known that the bond between the professional, child, and family encourages adherence to consultations and strengthens confidence in the interventions performed by the nurse [26]. In a study conducted in the Midwest region of Brazil, nurses highlighted the ease of establishing this bond, given that child monitoring begins as early as the prenatal period [27], reinforcing the importance of longitudinal care. In this sense, standardized documentation assists in information retrieval, which is essential for understanding the child and family context.

The number of subset nursing interventions identified in the consultations suggests a limitation in its applicability. One hypothesis for this limitation may be linked to the fact that several interventions are repeated across different diagnoses or are highly specific to a particular diagnosis.

Consideration must also be given to the configuration of the observed context, which interferes with the presence of certain interventions. In the PHC units that served as research settings, almost all evaluated children presented adequate development. It is recommended that future studies, preferably multicenter ones, advance the applicability of the proposed subset by expanding its use in different care and sociocultural contexts. Conducting studies with more heterogeneous populations regarding children’s health conditions, family contexts, sociodemographic characteristics, and levels of social vulnerability is encouraged to evaluate the applicability, representativeness, and stability of the elements that comprise the subset across distinct pediatric healthcare settings. Expanding the diversity of the investigated populations may strengthen the evidence regarding the subset’s applicability.

Another element influencing the low identification or observation of the subset’s nursing interventions stems from the fact that the presence of the nursing diagnosis “Child development, effective” automatically excludes nursing diagnoses related to potential risks or negative health conditions and, consequently, their corresponding nursing interventions. It is suggested that future studies analyze nursing consultations focused on children with developmental delays or alterations to verify the applicability of nursing diagnoses related to risks and negative health conditions, along with their respective nursing interventions.

In the studied scenario and within the routine of well-child consultations in PHC, the prevalence of risk conditions or child development alterations was low. However, this reality does not reflect the whole Brazilian territory, where social determinants and inequalities are responsible for different child development outcomes. A study evaluating socioeconomic, family, and individual factors associated with child development in the first year of life among socially vulnerable families—including 3242 children residing in 30 municipalities across Brazil’s five regions—concluded that potentially modifiable characteristics, such as maternal education, maternal depression, and prematurity/intrauterine growth restriction, exerted the greatest impact on reducing child development scores [28]. The use of resources that enhance the monitoring of conditions promoting child development and risk reduction, such as the evaluated subset, is essential to strengthen the nurse’s proactivity in identifying vulnerable clientele in their territory and offering access to nursing consultations.

The results suggest that the low frequency of the subset’s nursing interventions may have been influenced by the organization of access to PHC services in the PHC units across both research settings. Aspects such as available time, patient workflow, and prioritization of immediate demands can limit the execution of interventions provided in the subset and compromise the quality of care delivered. This finding corroborates results from a study conducted in the state of Rio de Janeiro, which demonstrate that the quality of nursing care is directly linked to working conditions and service organization [29]. Furthermore, the lack of integration of the subset into the electronic health record may have hindered its systematic incorporation into practice and subsequent nursing documentation. Thus, the low frequency observed should not be interpreted exclusively as an absence of care, but as a possible reflection of documentation practices, service organization, and the need to review the adequacy of these interventions within protocols and systems.

The omission of the follow-up consultation recommended by the applicability analysis method is considered a limitation of this study. The timeline of the research schedule did not allow for this step of the method to be carried out, which may have affected the overall nursing diagnoses, outcomes, and interventions. In addition, the study presents limitations related to convenience sampling and the number of participants—limitations identified by the method’s authors that should be addressed in future research—which does not compromise the quality of the results obtained.

The use of a single observer, without formal measurement of data agreement by the observed nurse, and their presence during consultations may have introduced subjectivity and the Hawthorne effect. These limitations were partially mitigated by observing consultations under routine care conditions, requesting verification of the observed information, and utilizing a structured instrument alongside standardized observation criteria.

The predominance of healthy children, gaps in certain age groups, the criterion of considering practice performed based on a single occurrence, and the absence of a follow-up consultation may have limited the identification of more complex interventions or those delivered over time. Nevertheless, the novelty of this study focused on the applicability of subsets in PHC offers an exploratory overview of child development promotion practices, identifies care gaps, and provides insights for broader and longitudinal future research.

## 5. Conclusions

The findings of this study support the terminological subset as a valuable tool to standardize and improve the quality of clinical nursing records, while indicating the need for additional evaluations in scenarios involving children at greater risk or with increased vulnerability. Furthermore, these findings contribute to analysis within computerized contexts.

A total of 11 nursing diagnoses/outcomes (64.7%) and 13 nursing interventions (21.3%) from the ICNP^®^ Subset for Child Development Monitoring in children aged zero to three years were considered clinically applicable. The identified or observed nursing interventions addressed different nursing diagnoses, reflecting the most frequently utilized interventions in conducting childcare consultations with positive nursing diagnosis.

Data collection conducted in two Brazilian regions with distinct sociodemographic characteristics provides initial evidence of the potential use of the terminological subset for monitoring the development of children aged zero months to 3 years in PHC. Furthermore, it paves the way for expanding investigations to other regions of the country or global settings where PHC nursing practice is similar to that in Brazil. Future research is expected to promote a more robust analysis of the subset and contribute to the consistency of performing and documenting nursing consultations, enabling access to data on the impact of nursing actions in promoting child development.

Multicenter studies including a larger number of participants and observing follow-up consultations could strengthen the evidence and verify the contribution of using the subset for continuity of care, the measurement of nursing-sensitive outcomes, and the early identification of changes in child development.

## Figures and Tables

**Table 1:** Nursing diagnoses and outcomes (positive and risk-related) identified or observed during nursing consultations. Paraná; São Paulo (2025).

Nursing Diagnoses/ Outcomes (Positive)	Nursing Diagnoses/ Outcomes (Risk-Related)
Effective child development	Risk for impaired child development
Caregiver attention Effective psychomotor activity Effective communication ability Effective cognitive learning Effective personal–social ability	Risk for impaired parenting ability Risk for impaired psychomotor activity Risk for impaired communication Risk for impaired personal–social ability

**Table 2:** Absolute and relative frequency of the nursing diagnoses by area in the observed consultations, categorized by city (*N* = 46). São José dos Pinhais; São Paulo, 2025.

Diagnoses	Area	Paraná (*n*)	São Paulo (*n*)	%
Effective psychomotor activity	Motor	20	24	95%
Effective communication ability	Language	20	23	93%
Effective personal–social ability	Personal–social	20	8	60%
Effective cognitive learning	Adaptive	7	3	21%

**Table 3:** Absolute and relative frequency of nursing interventions identified or observed in 20 or more nursing consultations (*N* = 46). Paraná; São Paulo, 2025.

Interventions	Consultation (*n*)	%
Planning a new appointment for child development assessment	37	80%
Informing the mother/caregiver about the assessment result	36	78%
Praising the mother/caregiver	35	76%
Reinforcing with the mother/caregiver how to stimulate the child	27	58%
Informing the mother/caregiver about the importance of play for the child	24	52%
Documenting information about the child	22	47%
Applying a child development assessment instrument	21	45%

## Data Availability

The original data presented in the study are openly available in Zenodo at https://doi.org/10.5281/zenodo.20797901.

## Abbreviations

PHC	Primary Health Care
ICNP^®^	International Classification for Nursing Practice
ICN	International Council of Nurses
SNOMED-CT	Systematized Nomenclature of Medicine—Clinical Terms
UBS	Basic Health Units
G1	Group 1
G2	Group 2
